# Race Differences in Trajectories of Hopelessness Among U.S. Older Adults: Do Social Conditions Matter?

**DOI:** 10.1093/geroni/igab046.989

**Published:** 2021-12-17

**Authors:** Uchechi Mitchell, Elena Graetz, Jing Wang

**Affiliations:** 1 University of Illinois Chicago, School of Public Health, Chicago, Illinois, United States; 2 University of Illinois Chicago, College of Liberal Arts and Science, University of Illinois, Chicago, Illinois, United States; 3 University of Illinois, Chicago, University of Illinois Chicago, Illinois, United States

## Abstract

Despite being a risk factor for cardiovascular disease, suicidal ideation, and mortality among U.S. older adults, research on hopelessness and how it changes over time are lacking. Although hopelessness generally increases with age, levels of hopelessness may be influenced by race/ethnicity and social or economic factors. This study uses longitudinal data from 8,359 individuals from the Health and Retirement Study to examine race differences in trajectories of hopelessness from 2006 to 2018. We used linear mixed models to estimate trajectories of hopelessness for blacks, whites and Hispanics age 51 and older. The model was fit with a natural spline cubic function to model changes in time trends of hopelessness and the interaction between time and race. Models controlled for demographic characteristics, socioeconomic status, health status, and psychosocial factors that influence hopelessness. We found that older Hispanics have the highest levels of hopelessness, followed by non-Hispanic blacks and non-Hispanic whites. Trajectories of hopelessness were non-linear and differed by race. For older whites, hopelessness increased from 2006-2010 and then decreased until 2018. For older blacks, it decreased the entire time period but did so at a decreasing rate; and, for older Hispanics, hopelessness decreased from 2006-2012 and then increased thereafter. Our study shows that hopelessness generally decreased over time among older adults between 2006 and 2018 in race-specific ways, despite generally increasing with age. These findings suggest that race, age and period effects differentially influence trajectories of hopelessness. Factors contributing to these differences may be related to concurrent social and economic conditions.

